# Reducing Nitrogen Dosage in *Triticum durum* Plants with Urea-Doped Nanofertilizers

**DOI:** 10.3390/nano10061043

**Published:** 2020-05-29

**Authors:** Gloria B. Ramírez-Rodríguez, Cristina Miguel-Rojas, Gabriel S. Montanha, Francisco J. Carmona, Gregorio Dal Sasso, Josefina C. Sillero, Jan Skov Pedersen, Norberto Masciocchi, Antonietta Guagliardi, Alejandro Pérez-de-Luque, José M. Delgado-López

**Affiliations:** 1 Department of Inorganic Chemistry, Faculty of Science, University of Granada, Av. Fuente Nueva, s/n, 18071 Granada, Spain; gloria@ugr.es; 2 Department of Science and High Technology and To.Sca.Lab, University of Insubria, Via Valleggio 11, I-22100 Como, Italy; cristinademiguelrojas@gmail.com (C.M.-R.); fjcfer@gmail.com (F.J.C.); norberto.masciocchi@uninsubria.it (N.M.); 3 IFAPA Alameda del Obispo, Area of Genomic and Biotechnology, Avenida Menéndez Pidal, S/N, 14004 Córdoba, Spain; josefinac.sillero@juntadeandalucia.es; 4 Center of Nuclear Energy in Agriculture (CENA), University of São Paulo (USP), Avenida Centenário 303, 13416-000 Piracicaba, São Paulo, Brazil; gabriel.montanha@usp.br; 5 Institute of Crystallography and To.Sca.Lab, Consiglio Nazionale delle Ricerche (IC-CNR), Via Valleggio 11, I-22100 Como, Italy; gregorio.dalsasso@ic.cnr.it (G.D.S.); antonella.guagliardi@ic.cnr.it (A.G.); 6 Department of Chemistry and Interdisciplinary Nanoscience Center (iNANO), Aarhus University, Gustav Wieds Vej 14, 8000 Aarhus, Denmark; jsp@chem.au.dk

**Keywords:** nanofertilizer, agriculture, calcium phosphate, wheat, quality, urea

## Abstract

Nanotechnology is emerging as a very promising tool towards more efficient and sustainable practices in agriculture. In this work, we propose the use of non-toxic calcium phosphate nanoparticles doped with urea (U-ACP) for the fertilization of *Triticum durum* plants. U-ACP nanoparticles present very similar morphology, structure, and composition than the amorphous precursor of bone mineral, but contain a considerable amount of nitrogen as adsorbed urea (up to ca. 6 wt % urea). Tests on *Triticum durum* plants indicated that yields and quality of the crops treated with the nanoparticles at reduced nitrogen dosages (by 40%) were unaltered in comparison to positive control plants, which were given the minimum N dosages to obtain the highest values of yield and quality in fields. In addition, optical microscopy inspections showed that Alizarin Red S stained nanoparticles were able to penetrate through the epidermis of the roots or the stomata of the leaves. We observed that the uptake through the roots occurs much faster than through the leaves (1 h vs. 2 days, respectively). Our results highlight the potential of engineering nanoparticles to provide a considerable efficiency of nitrogen uptake by durum wheat and open the door to design more sustainable practices for the fertilization of wheat in fields.

## 1. Introduction

According to the Food and Agriculture Organization (FAO) of the United Nations, the world population is expected to increase to almost 10 billion by 2050 [1]. Considering the limited land availability, the increased frequency of extreme climate events, and the current inefficient utilization of resources (e.g., water, energy, and nutrients), meeting the future global food demand sustainably is one of the major challenges that agriculture, and the whole food supply chain, need to face in the future [2,3]. The intensive application of conventional agrochemicals has already been demonstrated to result in an unsustainable impact on the environment [4]. Nitrogen, the most important nutrient for crop production, plays the major role in agriculture, when the energy spent for its synthesis, the tonnage and the monetary value are, all together, taken into account [5]. Nevertheless, 50–70% of N applied through conventional fertilization is lost in the form of either water soluble nitrates, gaseous ammonia, and nitrogen oxides, or incorporated as minerals into the soil by microorganism-mediated activity [6,7]. The intensive application of N and P fertilizers over the last 40 years has indeed become one of the major anthropogenic factors in the eutrophication, groundwater contamination, and caused important changes in the soil chemistry and microbial communities [7]. Thus, new pathways to increase crop yield and quality while mitigating the farming environmental impact are needed [8].

In this scenario, nanotechnology is offering great promises for the safe and efficient delivery of agrochemicals [3,9]. The use of nanoparticles to specifically release nutrients inside the plant can minimize their losses, avoiding rapid changes in their chemical nature. Inorganic, organic, and composite nanomaterials have been tested on different plants to assess their potential impact on plant growth, development, and productivity [5,10,11,12]. However, despite the fact that N is the most important nutrient for crop production, there are still few studies only reporting on nanomaterials supplying this nutrient, as recently pointed out by Kopittle et al. [13]. Interesting results were obtained when nanocrystals of hydroxyapatite [HA, Ca_5_(PO_4_)_3_OH], the main constituent of hard tissues, and one of the most widely used biomaterials in medicine [14], were functionalized with urea [15,16,17]. The slow and gradual release of nitrogen from these urea-HA nanohybrids resulted in increased rice crop yields at a 50% lower dosage of urea in comparison to control crops treated with crystalline urea [17]. In a recent work [18], some of us incorporated urea (and other macronutrients) on amorphous calcium phosphate nanoparticles (ACP), the mineral precursor of HA [19]. Its amorphous nature entails a higher capacity to incorporate foreign ions, higher adsorption capacity, and higher solubility than HA [18]. We observed that the nanoparticles provided a gradual release of urea, concomitantly with the ionic components (calcium, phosphate, and potassium) and provided good grain yields at reduced nitrogen rates [18]. 

Nitrogen fertilization also has a great impact on crop quality [20], which, in turn, affects the price and the final incomes for farmers [21]. Nonetheless, the footprint that nanoparticles have on crop quality has not yet been evaluated [20]. The present work aims to demonstrate the capability of urea-doped ACP nanoparticles (U-ACP) to maintain yields and quality of the crops simultaneously at reduced nitrogen dosages. After a complete characterization of the size, structure, and composition of the nanoparticles, plant tests were performed on durum wheat (*Triticum durum* L) under controlled conditions (growth chamber). Relevant yield parameters (i.e., shoot, ear, kernel numbers, and weights) and quality indicators (i.e., protein content and vitrousness), were assessed after plant growth was completed. We found out that both yields and quality of the crops treated with the nanoparticles were unaltered in comparison to control plants, to which much higher nitrogen dosages were supplied [22,23]. Preliminary experiments with stained ACP nanoparticles were also carried out to gain insights on the routes of nanoparticle uptake and on their translocation in plant tissues.

## 2. Materials and Methods 

### 2.1. Materials

Calcium nitrate tetrahydrate (Ca(NO_3_)_2_·4H_2_O, ≥99.0% pure, BioXtra), sodium citrate tribasic dihydrate (Na_3_(C_6_H_5_O_7_)∙2H_2_O, ≥99.0% pure (Na_3_(Cit)), urea (pellets, ≥99.5%, ReagentPlus^®^), potassium phosphate dibasic anhydrous (K_2_HPO_4_, ≥99.0% pure), sodium carbonate (Na_2_CO_3_, ≥99.0% pure, BioXtra), and potassium nitrate (KNO_3_, ≥99.0%), were purchased from Sigma Aldrich (Madrid, Spain). All the solutions were prepared with ultrapure water (0.22 µS, 25 °C, MilliQ^©^, Millipore, Merck, Darmstadt, Germany).

### 2.2. Synthesis of U-ACP Nanocomposites

The synthesis was carried out through a simple batch method, which does not require hazardous reagents. It consists on mixing 100 mL of an aqueous solution (A) containing 0.2 M Ca(NO_3_)_2_, 0.2 M Na_3_Cit and 8 g of urea with a solution (B) of an equal volume containing 0.12 M K_2_HPO_4_ and 0.1 M Na_2_CO_3_. The mixture was then kept at 37 °C for 5 min. After that, the precipitates were repeatedly washed with ultrapure water by centrifugation (5000 rpm for 15 min), and then freeze-dried (Cryodos lyophilizer, Telstar) overnight under vacuum. 

### 2.3. Characterization of the Nanomaterial

The morphology and composition was analyzed by transmission electron microscopy (TEM). Nanoparticles were dispersed in pure ethanol, deposited on 200 mesh copper grids covered with thin amorphous carbon films. TEM images were collected with a 300 kV FEI TITAN G2 60–300 microscope (Thermo Fisher Scientific, Waltham, MA, USA) of the Centre for Scientific Instrumentation, University of Granada (CIC-UGR). Nanoparticle size distribution (mean diameter and standard deviation) was estimated by measuring the diameter of 100 nanoparticles with ImageJ software (version 1.48v; NIH, Bethesda, MD). Scanning transmission electron microscopy (STEM) images were acquired with a HAADF detector. The elemental composition of selected areas in STEM mode was determined by energy dispersive X-ray spectroscopy (EDS) with a quad-silicon drift detector (Super-X/ChemiSTEM). Images were analyzed with the Velox software (Thermo Fisher Scientific, Waltham, MA, USA).

Small-Angle X-ray scattering (SAXS) measurements were performed on the in-house instrument at Aarhus University [24], which uses a rotating Cu anode source. The beam is collimated and focused by side-by-side Montel multilayer mirrors and the scattered X-rays are collected by a Vantec 500 detector (Bruker AXS, Karlsruhe, Germany). The collimation system consists of two-pinholes, where the one close to the sample is a scatterless pinhole with edges of Ge crystal [25,26]. The sample powders were mounted by picking up a thin layer of each sample by matte acetate Scotch tape. The samples were then mounted in the beam in the integrated vacuum of the SAXS instrument and a piece of the same tape was measured and subtracted as background. The scattering intensity (*I(q)*) was plotted as a function of the scattering vector, *q* = 4πsin(θ)/λ, being 2θ the scattering angle and λ the operational wavelength (1.5406 Å). The in-house developed SUPERSAXS program package (C.L.P. Oliveira and J.S. Pedersen, J.S., unpublished) was used for the data treatment. The errors from counting statistics were used in the weighted least-squares fits to the experimental data; a constant background was included in the fits to account for any error in the background subtraction. The data could be described by a model for polydisperse cylindrical, circular disks. The angular integration over orientation was carried numerically and the polydispersity was included in an external loop with a Schulz number distribution, and the polydispersity was assumed to be affine. 

X-ray powder diffraction (XRPD) patterns of the samples were recorded on a D8 Advance diffractometer (Bruker AXS, Karlsruhe, Germany) equipped with a Lynx-eye position sensitive detector using Cu Kα radiation (λ = 1.5418 Å) generated at 40 kV and 40 mA. XRPD patterns were recorded in the 2θ range from 10 to 60° with a step size Δ2θ of 0.021 and a counting time of 0.5 s/step.

Fourier transform infrared (FTIR) spectra were collected on a Tensor 27 (Bruker, Karlsruhe, Germany) spectrometer by accumulation of 25 scans in the 4000–400 cm^−1^ range with a resolution of 2 cm^−1^. 

The chemical composition of powdered samples (Ca and P) was analyzed by inductively coupled plasma optical emission spectrometry (ICP-OES, Optima 8300, Perkin-Elmer Inc., Waltham, MA, USA). 20 mg of the powdered sample were dissolved in 2 mL of ultrapure nitric acid and then diluted up to 100 mL with Milli-Q water. The emission wavelengths were 317.93 nm (Ca), 213.62 nm (P), and 766.49 nm (K). The total nitrogen content of the U-ACP nanocomposite was measured by elemental analysis with a Thermo Scientific Flash 2000 Organic Elemental Analyzer equipped with a microbalance (XP6, Mettler Toledo, Columbus, OH, USA) of the CIC-UGR. The urea content of U-ACP was quantified using the p-dimethylamino-benzaldehyde colorimetric method [27]. To this aim, 10 mg of powdered U-ACP sample was dispersed in 1 mL of ultrapure water and after 72 h, the sample was centrifuged and the urea concentration of the supernatant was measured by UV−vis spectroscopy [18]. Carbonate, citrate, and water contents were determined by thermogravimetry as described in [28].

### 2.4. Experiments on Durum Wheat under Controlled Conditions 

The efficiency of U-ACP nanoparticles as nitrogen nanofertilizer was tested on wheat under controlled conditions (growth chamber), using the commercial variety of durum wheat (*Triticum durum*) Amilcar (Geslive, Sevilla, Spain). One durum wheat seed was sowed in each 113 cm^2^ plastic container (plant pots) filled with a non-sterile 1:1 soil/sand mixture, and cultivated in a growth chamber at 22 ± 1 °C, illuminated with simulated sunlight (12:12 h light/dark cycle). The soil was collected from Santaella, Southern Spain (province CO Lat. 37°34’03´´N; Long. 4°50′48´´O), which has clay loam soil type. 

Three nitrogen-fertilizing treatments were used in this study with a completely randomized duplicated block design with 12 plants per block (12 pots × 2 blocks × 3 treatments). An initial amount of diammonium hydrogenphosphate (DAP, 36 kg of N ha^−1^) was applied to all the treatments at planting. Tap water was applied as irrigation water when needed, usually three times a week. During the stem elongation, and just before ear formation, the three groups of plants were treated as follows: (1) untreated control group, receiving only water; (2) U-ACP fertilization group, receiving 15 kg of N ha^−1^ as sprayed aqueous suspension of U-ACP (over the trays) and 60 kg of N ha^−1^ in the form of granular DAP in the soil; (3) positive control, receiving double nitrogen dosage in total, i.e., 150 kg of N ha^−1^ in the form of granular DAP in the soil. The nitrogen dosage of the positive control was based on previous field experiments, which demonstrated that rates of 150 kg of N ha^−1^ are needed to obtain the highest values of yield or quality [22,23]. The total nitrogen dosage with the nano-treatment was then reduced by ca. 40% with respect to the positive control (i.e., 111 vs. 186 kg N ha^−1^ in total, respectively). Thus, the discussions on nitrogen reduction are referred to the total nitrogen applied to the plant in each treatment. 

Control experiments with 60 kg of N ha^−1^ in the form of granular DAP or 60 kg of N ha^−1^ in the form of granular DAP complemented with 15 kg of N ha^−1^ in the form of aqueous solutions of DAP were not carried out since those nitrogen dosages of highly soluble fertilizers would result in a dramatic reduction of yields and quality of the crops, as observed in previous field tests on durum wheat [22,23].

The above-ground biomass and seeds were completely dried and weighed once the final plant physiological maturity was reached. Kernel number was determined using a seed counter machine (Sadkiewicz Instruments, Warsaw, Poland), while hard vitreous kernel was determined using a Pohl Farinator (Bipea Reference method 204–1104). Protein content was quantified by the standard Kjeldahl method [29,30].

### 2.5. Nanoparticles Uptake and Localization in Wheat Plants

Seeds of durum wheat (*T. durum*) cv. Amilcar (Geslive, Sevilla, Spain) were sown in pots with a mix of commercial compost (Suliflor SF1 substrat; Suliflor Lithuania) and sand. The plastic pots were placed in trays containing water and maintained in a growth chamber under the same conditions as described above.

Alizarin Red S (VWR Life Science) solution, a specific dye for histochemistry of calcified animal and plant tissues, was prepared at 2% (v/v) for staining U-ACP nanocomposites. For this purpose, 2 g of Alizarin Red S were diluted in 100 mL of Milli-Q^®^ water. The resulting content was filtered using a 0.22 µm MF-Millipore MCE membrane (MILLEX^®^GS). The solution was kept in an amber flask at room temperature. The nanocomposite dispersion was prepared at 10 mg mL^−1^. For this, exactly 10 mg of the synthesized nanoparticles was weighed in an analytical balance (Denver Instrument, Bohemia, NY, USA), and 20 µL of the 2% Alizarin Red S staining solution was added. After one hour, the content was transferred to a 2 mL Eppendorf^®^ plastic vial, and its volume was completed to 1 mL using Milli-Q^®^ water. Subsequently, the content of the vials was homogenized using a test-tube shaker (Heidolph REAX 200, Heidolph Instruments GmbH & CO. KG, Schwabach, Germany) for 2 min.

Droplets of the nanocomposite dispersions were exposed to wheat roots and leaves, and the fresh tissues were assessed through optical microscopy. For root application, the plants were carefully removed from the pots, and repeatedly washed with deionized water. Afterward, the plants were placed in a tray containing a wet paper, and 5 µL droplets were applied on located places. After one hour, root samples from the inoculation points were detached, washed with deionized water, and hand-cut cross sections obtained using a chirurgic steel blade. Sections were immediately placed into a glass slide and mounted with a coverslip and deionized water, subsequently analyzed using a Nikon Eclipse 50i optic microscope (Nikon Instruments Inc., Melville, NY, USA). For the foliar application, the pots were placed horizontally into a tray, leaves were carefully extended and fixed on the tray bottom using cello tape, and 5 µL droplets were applied using a micropipette on located places along each leaf. The plants remained laid for 150 min for the droplet drying, and subsequently, were placed back into vertical position, transferred to a tray containing water, and maintained in the growth room for 48 h. Afterward, the wheat leaves were detached, and the spots where the droplets were applied were sampled by cutting with a chirurgic steel blade. The sampled leaf tissues were immediately washed using deionized water and transferred to a 96% ethanol solution for 48 h to remove chlorophyll. Then, samples were washed using deionized water and immediately mounted on a glass slide with glycerol solution 50% (v/v) and a coverslip. The slides were observed using the same microscope described above. Images were taken with lenses from the Plan Fluor series by Nikon Instruments Inc., through a Nikon DS-Fi1 digital optic device and connected to a PC through the Nikon DS-U2 control unity (Nikon Instruments Inc., Melville, NY, USA). Negative controls were prepared using water. Positive controls were also prepared by applying only Alizarin Red S solutions to confirm the absence of dye-related artifacts.

### 2.6. Statistical Analyses 

For all the assays, every treatment was replicated three times. All the experiments were conducted using a randomized experimental design. For the wheat experiments, three blocks (20 plants per block, thus 60 seeds altogether in one treatment) were set for each treatment using a randomized design. Statistical analysis of the data was performed using Statistix 9.0 (Analytical Software, Tallahassee, FL, USA). Data for 1000 kernel weight, kernel number, hard vitreous kernel, and protein content were processed using the analysis of variance (ANOVA). Differences between means were compared using the least significant difference (LSD), fixing at 0.05 the significance level.

## 3. Results and Discussion

### 3.1. Synthesis and Characterization of U-ACP Nanocomposites

**Scheme 1:**
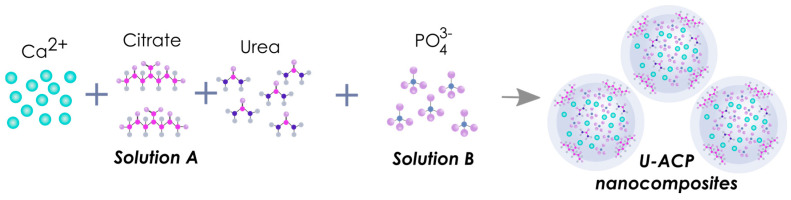
Graphical sketch of the batch method used to obtain U-ACP nanofertilizers.

**Figure 1:**
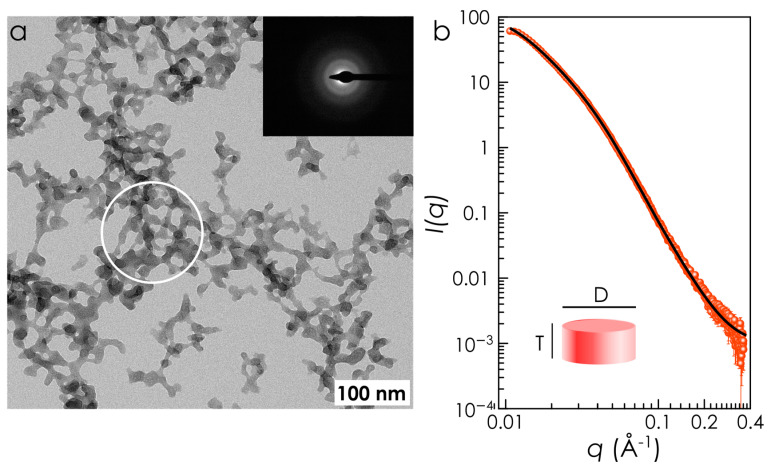
Morphological characterization of U-ACP nanocomposites. (**a**) TEM micrograph of U-ACP nanoparticles. The amorphous nature of the particles is confirmed by the lack of diffraction spots in the selected-area electron diffraction (SAED) pattern (inset). SAED pattern was collected on the particles under the white circle. Mean particle diameter of 13.8 nm and dispersion (standard deviation) of 4.2 nm were obtained after measuring 100 nanoparticles. (**b**) SAXS data (log-log scale, symbols) and best fitting (line) obtained with analytical form factors of a finite disk-shaped model (inset). The analysis provided number-based mean diameter, <D>_N_ = 13.5 (3) nm, and thickness, <T>_N_ = 3.46 (6) nm.

The batch method to produce urea-functionalized amorphous calcium phosphate nanoparticles (U-ACP) is depicted in Scheme 1. It consists in mixing a solution containing calcium, citrate (an important component of bone organic matrix [28,31]) and urea with a phosphate—containing solution at 37 °C and atmospheric pressure. The precipitation of irregularly shaped amorphous nanoparticles with an average diameter of 13.8 nm (Figure 1a) suddenly occurs after mixing. The nanoparticle sizes and shapes were analyzed in much more detail by small-angle X-ray scattering (SAXS), capable of providing more accurate and statistically significant size distributions (Figure 1b). The best fit of our SAXS data was obtained by adopting a cylindrical, though oblate, model for disk-shaped nanoparticles, 13.5 (3) nm in diameter and 3.46 (6) nm thick (aspect ratio = 0.26) with a relative dispersion of 0.61 (Figure 1b). The average diameter of the nanoparticles as extracted by TEM and SAXS are in perfect agreement. 

**Figure 2:**
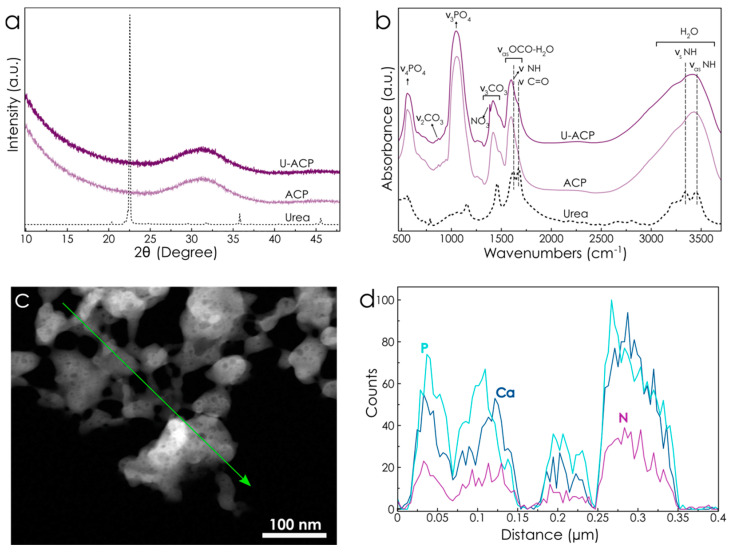
Structural and compositional characterization of U-ACP. XRD patterns (**a**) and FTIR spectra (**b**) of crystalline urea (dotted line), non-doped ACP nanoparticles and U-ACP nanocomposite. High-angle annular dark-field (HAADF) image (**c**) and line profile energy-dispersive X-ray spectroscopy (EDS) of U-ACP nanoparticles showing the Ca Kα (dark blue curve), P Kα (light blue), and N Kα (magenta) signals (**d**).

It is well known that during urea-doping of nanoparticles, an excess of (non-bonded) urea can precipitate as micro-crystalline phase during drying; this unintentional result has indeed been previously obtained during urea-coprecipitation syntheses [15,17]. However, the absence, in the XRPD pattern, of Bragg peaks assignable to crystalline urea (and specifically of the sharp and intense diffraction peak at ca. 22° 2θ for Cu-Kα radiation, the 110 reflection of crystalline urea) confirms the fully amorphous nature of U-ACP and definitively rules out the formation of a biphasic conglomerate material, where ACP and crystalline urea are spatially segregated (see Figure 2a).

The FTIR spectrum of U-ACP shows absorption vibrational bands of urea at ca. 1627, 1677, 3340, and 3440 cm^−1^ ascribed to N-H bending (δNH), carbonyl (νC=O) stretching and N—H stretching modes (νNH), respectively, in addition to the typical phosphate absorption bands of undoped nanoparticles (ACP) (Figure 2b) [18,32]. Both spectra additionally display absorption bands associated with carbonate, citrate, and water (Figure 2b), all of them important components of bone mineral. Further evidences of the hybrid nature of the nanoparticles were provided by energy-dispersive X-ray spectroscopy (EDS) on individual U-ACP nanoparticles (Figure 2c,d). The nanoparticles are composed of calcium, phosphorous and nitrogen (Figure 2d), and the spatial correlation between the Ca/P/N EDS peak intensities confirms the substantial amount of urea bound to the ACP nanoparticles, and not located elsewhere.

The chemical analysis of powdered nanoparticles confirmed the significant amount of Ca (22.9 ± 0.1 wt %), P (10.1 ± 0.1 wt %), and N (3.0 ± 0.3 wt %) (Table 1). The nanoparticles contain ca. 5.8 wt % of urea, which is then the main nitrogen source. Nonetheless, a residual amount of nitrogen as nitrate was found due to the use of Ca(NO_3_)_2_ as a calcium source during the synthesis (Figure 2b). The content of carbonate (6.0 ± 0.2 wt %), citrate (9.6 ± 0.2 wt %) and water (10.0 ± 0.3 wt %) (Table 1) was quantified by thermo-gravimetric analysis. The nanoparticles also contain 1.9 ± 0.2 wt % of K (other important plant macronutrient), being the rest oxygen. U-ACP nanoparticles are consequently very similar in size, composition, and structure to ACP, the amorphous precursor mineral of bone (for this so-called biomimetic), but with a considerable content of nitrogen. These biomimetic calcium phosphate nanoparticles are fully biocompatible, biodegradable, and do not present inherent toxicity [18,33], which explains their broad applicability in medicine [14,33,34,35]. These features, along with the capability of gradually release nitrogen and other nutrients [18], make these biomimetic nanoparticles the ideal candidate for a safer and more efficient delivery of nutrients in plants. 

### 3.2. Experiments on Durum Wheat under Controlled Conditions

**Figure 3:**
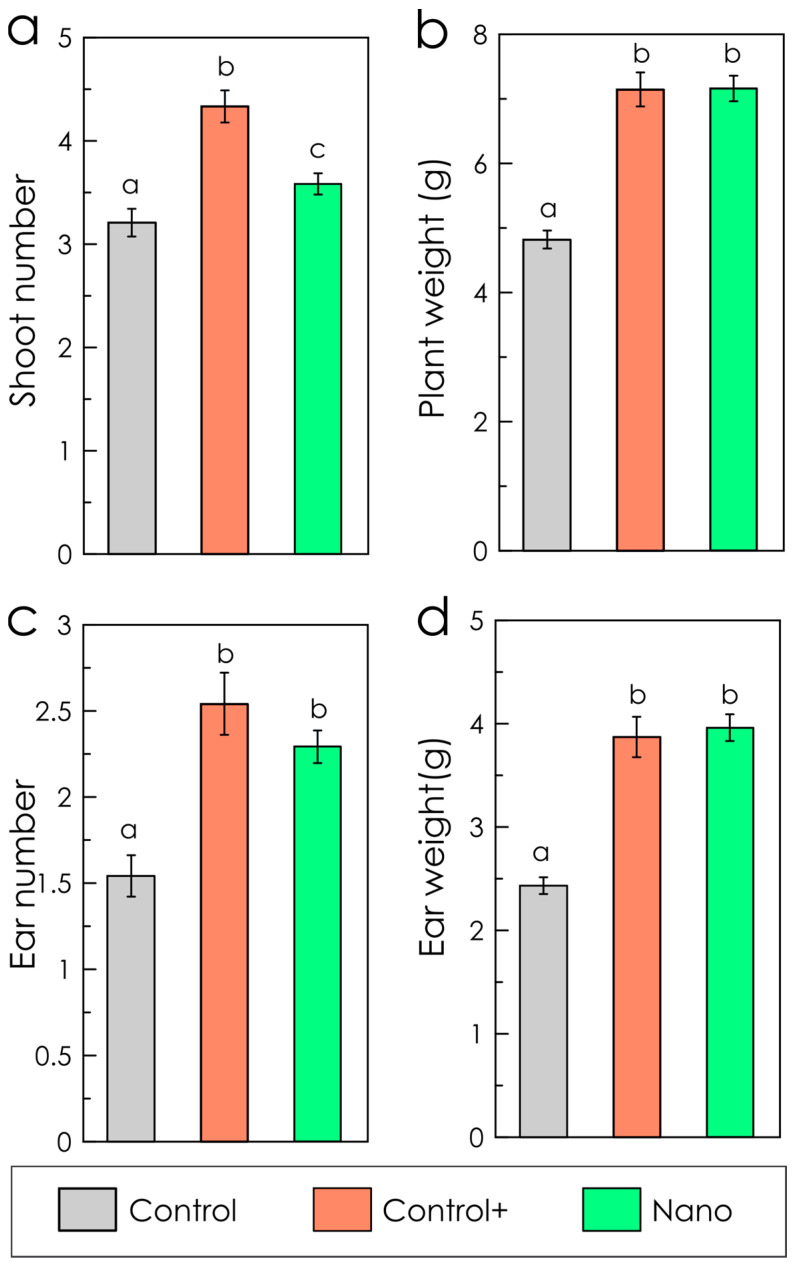
Plant growth and development parameters of durum wheat. Shoots number (**a**), average weight (**b**), ear number (**c**), and ear weight (**d**) per individual wheat plant. Data are expressed as mean ± standard error. Data with different letters are statistically different (*p* ≤ 0.05). Total Nitrogen dosages were 36 kg N ha^−1^ as granular DAP (control); 186 kg N ha^−1^ as granular DAP (positive control); and 111 kg N ha^−1^, of which 15 kg N ha^−1^ as sprayed aqueous suspension of U-ACP and 96 kg N ha^−1^ as granular DAP (nano-treatment).

The efficiency of the nanoparticles as a nitrogen supplier was tested on durum wheat [36], which is a very important crop in the Mediterranean Rim. It is used to obtain semolina, the raw material of pasta. [37]. The nitrogen rate of the nano-treatment was reduced by 40% with respect to the positive control. Nitrogen dosage of the latter was selected based on previous field experiments under Mediterranean conditions, which demonstrated that 150 kg of N ha^−1^ was the minimum rate to obtain the highest values of yield and quality [22,23]. Despite the considerable reduction of N content, plant growth, and development parameters (i.e., plant weight, ear number, and ear weight) of the nano-treatment and the positive control were comparable (Figure 3). However, these parameters were significantly reduced in untreated control plants, which received only tap water. Only tillering (shoot number, Figure 3a) of the positive-control was slightly higher, but those plants developed the same number of ears than plants treated with U-ACP. This finding suggests that N excess was wasted in producing vegetative growth with no effects on the final yield.

**Figure 4:**
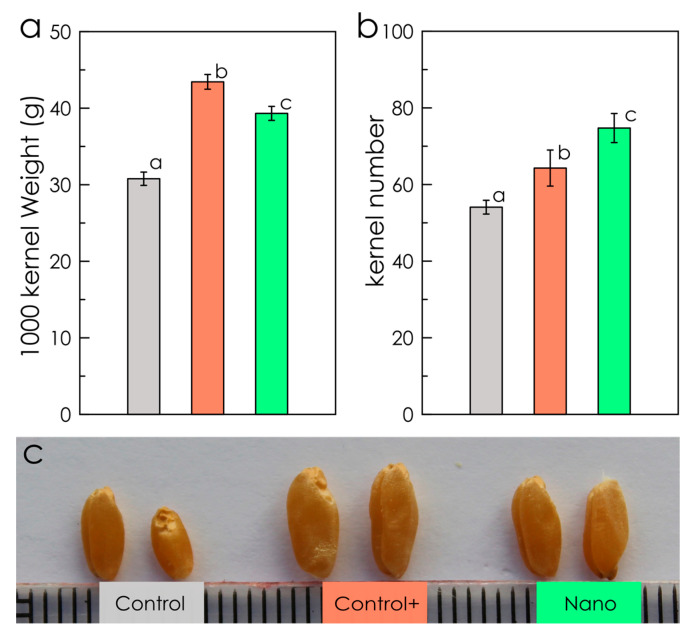
Yield parameters of durum wheat. Average thousand-kernel weight (**a**) and total kernel number (**b**) per plant. Data are expressed as average with the corresponding standard error as bars (*n* = 3). Different letters indicate significant differences between the treatments (*p* ≤ 0.05). Representative picture showing the kernel size of the different treatments (**c**).

In terms of yield, plants of the nano-treatment provided a higher number of smaller grains in comparison to the positive control (Figure 4), compensating size with the number, a well-known phenomenon in crops [38]. In fact, the total yield of all the plants at the end of the experiment for each treatment was 66.6 g and 65.5 g, respectively. These results indicate that the reduction of N with the nano-fertilization did not affect the final yield of the crops. Recent studies have shown that N availability can significantly influence the number of fertile florets and the proportion of those setting grains [39,40]. Our nano-treatment seems to favor this process, probably facilitating the absorption of N for the plant, which compensates for the reduction of applied nitrogen. As expected, control plants showed a sharp decrease in all the measured parameters (Figure 4), being the total yield of the plants 39.7 g. 

**Figure 5:**
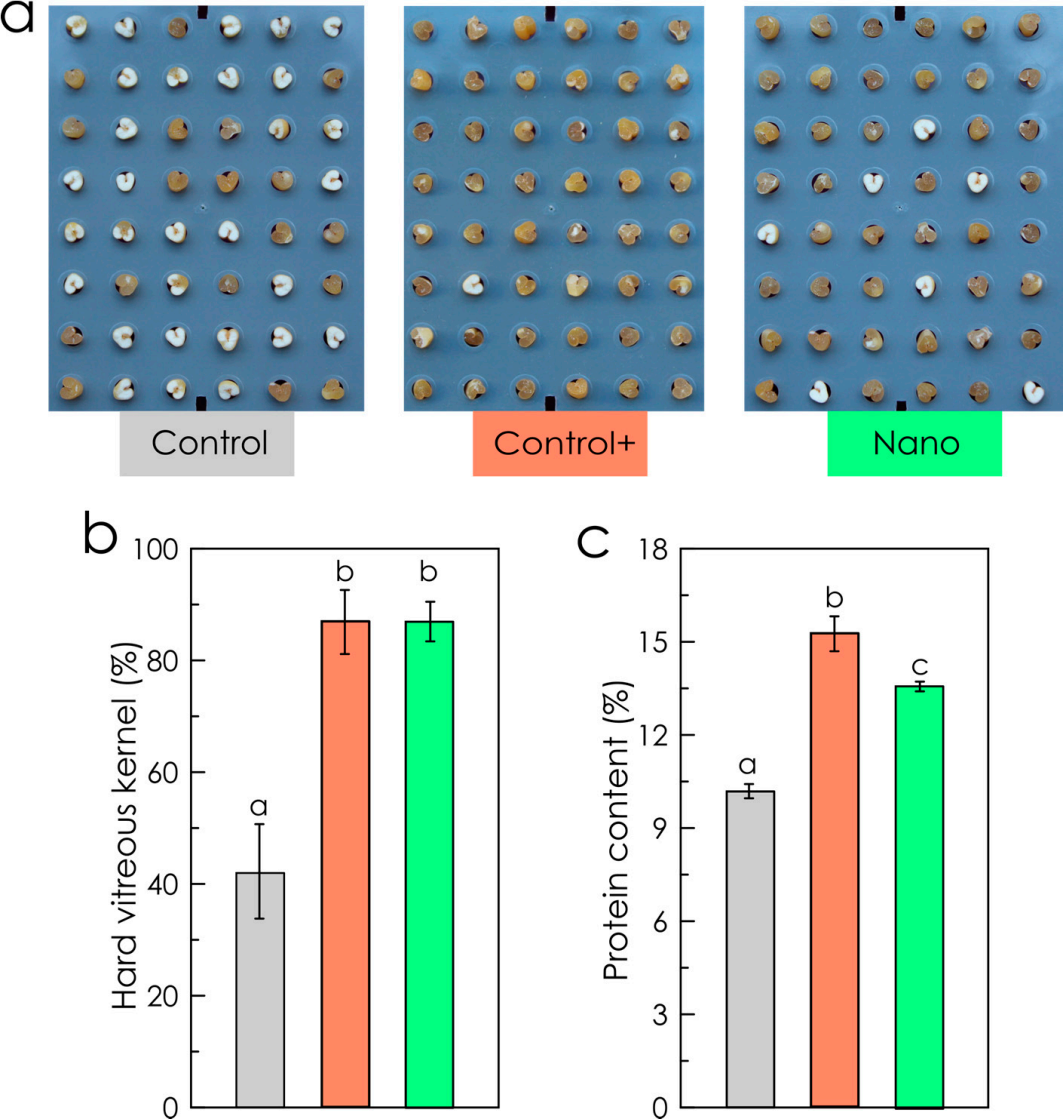
Quality parameters of durum wheat. Vitreousness of 48 split kernels (**a**). Percentage of hard vitreous kernel (**b**) and protein content (**c**) for the three treatments. Data are expressed as average with the corresponding standard error as bars (*n* = 3). Different letters indicate significant differences between the treatments (*p* ≤ 0.05).

The quality of the crops is also greatly influenced by the nitrogen rate of the fertilization. Concretely, N deficiency may cause important defects in the plant at the molecular level, which—particularly for durum wheat—affect the protein content and composition of the grain, directly linked to the development of hard vitreous kernels [41]. These parameters are indeed the most relevant indicators of the quality of the semolina extracted from the grains for pasta elaboration [42,43]. In our experiments, the hard vitreous kernels were practically the same for both fertilizations, appearing only 10% of the grains with a mealy aspect (white grains, Figure 5a,b). However, more than 50% of the grains of control plants showed such a mealy aspect (Figure 5a,b). The percentage of protein content was slightly reduced with the nano-fertilization but still remained above 13% (Figure 5c), the conventional threshold adopted for being classified within the higher quality group [44]. These results confirm that the important reduction of N did not affect the overall quality of the collected wheat grains. 

The finding here reported, along with very recent results on different crops and growth conditions [18,45,46], confirm the efficiency of engineered nanoparticles to deliver nitrogen in plants. Foliar application of U-ACP on Tempranillo grapevines (field experiments) resulted in a substantial increase of nitrogen use efficiency compared to conventional treatments (crystalline urea). Grapes treated with U-ACP at 0.4 kg N ha^−1^ contained similar yeast assimilable nitrogen (YAN) and amino acids (beneficial to wine aroma and taste) than those treated with crystalline urea at 15 times greater nitrogen doses (6 kg N ha^−1^) [45]. Additionally, fertilization tests on *Cucumis sativus L.* grown under hydroponic conditions have recently demonstrated that the use of U-ACP provides optimal root and shoot biomass and N content but with a 50% reduction of N dosage [46]. 

### 3.3. Nanoparticles Uptake and Localization in Wheat Plants

**Figure 6:**
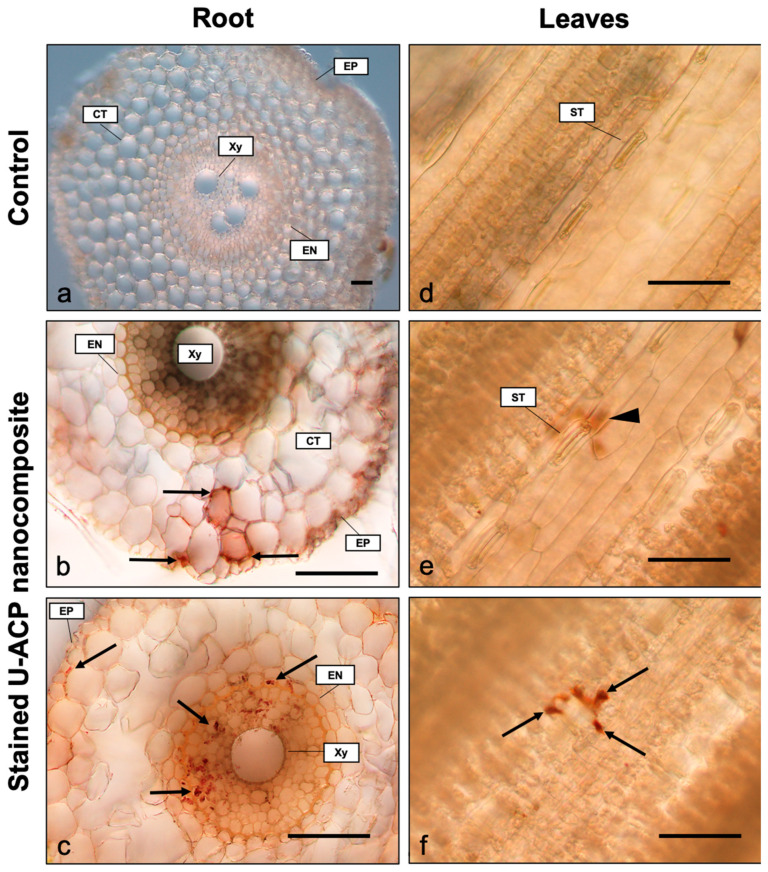
Histological localization of U-ACP nanocomposites inside wheat plants. Hand sections of wheat root (**a**,**b**,**c**) and leaf samples (**d**,**e**,**f**). Non-exposed controls show the absence of nanoparticles either in roots (a) and leaves (d). Accumulation of Alizarin Red S stained nanocomposites (arrows) can be observed in the apoplast of the root cortex (b) and inside the symplast of the central cylinder (c). In leaves, the presence of nanoparticles under the epidermis is first seen as an out of focus red-brownish stain (arrowhead) under the stomata (e), appearing as micro-sized aggregates (arrows) in the stomatal cavity when the plane is in focus (f). Note that (e) and (f) correspond to the same observation point with different focal planes. Scale bar = 50 µm. CT—cortex; Xy—xylem; EN—endodermis; EP—epidermis; ST—stomata.

Preliminary experiments were conducted to identify the routes of nanoparticles uptake and their further accumulation in plant tissues. To this aim, roots and leaves of treated plants were imaged by optical microscopy. The U-ACP nanocomposite was stained with Alizarin Red S, a specific dye for calcified animal and plant tissues [47,48]. In the roots, U-ACP nanocomposite penetrates the epidermis and seems to move radially through the cortex, following mainly the apoplastic pathway outside the cells (Figure 6b). On the other hand, once the nanoparticles reach the central cylinder, they follow a symplastic pathway and accumulate inside the cells (Figure 6c). This is likely due to the effect of crossing the endodermis, in which the materials are forced via symplast to cross the Casparian strip [49]. From this point, nanoparticles can be distributed to the whole plant through the xylem and phloem [49,50].

Considering the leaves, nanoparticle uptake seems to occur mainly through the stomata. Figure 6e shows the epidermis of a wheat leaf, and a red brownish coloration out of focus. When such stain is in the focal plane (Figure 6f), it corresponds to the accumulation of U-ACP nanocomposite under the epidermis, in the substomatal cavity, pointing towards the stomata as the main gateway for absorption. Nevertheless, internalization through the epidermis, as demonstrated in other cases [51], cannot be discarded, but it requires further investigations. Once the nanoparticles reach the substomatal cavity, they can translocate to other parts and organs of the plant, including neighboring leaves and the root [52,53].

Worth of note is the different absorption rate between nanoparticles applied to roots and leaves. Nanoparticles were observed inside the roots after 1 h of incubation, while 48 h were needed to detect accumulation inside the leaves. This can be an expected outcome, since the root is an organ specialized in the absorption of nutrients and water, and it contains specialized structures for that function such as root hairs, which increase the absorption surface. On the contrary, leaves main functions are photosynthesis and gas exchange, and despite water and nutrients also being able to pass through their tissues, they present some traits that acts as barriers for internalization, such as the cuticle (a waxy layer), which prevents water loss from the leaf surface, but also hampers penetration of substances through it. For that reason, only species able to cross the cuticle or penetrate through stomata (lacking cuticle coat) can be internalized in the leaves. In agreement with previous reports [54,55], our results confirm that nanoparticle surface dissolution (and consequently nutrient release) is not necessarily a limiting step to allow nutrients uptake through the leaves. This opens the door to the future development of ACP functionalized with other active species (besides fertilizers) for foliar applications. However, future efforts are needed to establish the best management practices for foliar treatment and, more importantly, to elucidate the uptake mechanism through the leaves and the translocation of the nanoparticles once inside. Interesting information can be then extracted to design novel nanoplatforms with higher level of performance. 

## 4. Conclusions

Amorphous calcium phosphate nanoparticles were successfully loaded with urea, the most widely used nitrogen fertilizer in agriculture on a global scale, eventually resulting in non-toxic nanoparticles which contain ca. 3 wt % of N. Experiments on *T. durum* plants under controlled conditions (growth chambers) demonstrated the efficacy of these U-ACP nanoassemblies to deliver urea. Interestingly, yield and quality parameters of the crops treated with the nanoparticles were similar to those treated with a conventional fertilization protocol (positive control), despite the nitrogen rate was reduced by 40% with the treatment containing nanoparticles. Preliminary studies with stained nanoparticles showed that the nanoparticles uptake takes place through both the stomata of the leaves and the epidermis of the roots. The uptake through the roots is much faster than through the stomata. Indeed, 1 h and 48 h were needed to detect the particles inside the roots and the leaves, respectively. Differences between radicular and foliar absorption and internalization pave the way for further field applications of ACP nanoparticles. 

The results reported here bring to light the possibility of using engineered nanoparticles to deliver nitrogen to plants more safely and efficiently. However, further research is still needed to secure the most suitable application protocols for real agricultural practices (field experiments). Following works should involve experiments with a wider range of concentrations, different application protocols and different crop species to gain a general overview of plant responses to treatments with ACP nanoparticles. The efficacy of foliar versus soil applications and the benefits of applying the nanoparticles at different plant growth stages should also be assessed. 

## Figures and Tables

**Table 1:** Chemical composition, wt %, of U-ACP nanoparticles. Data are expressed as mean ± standard deviation.

Ca ^a^	P ^a^	K ^a^	N ^b^	Carbonate ^c^	Citrate ^c^	Water ^c^
22.9 ± 0.1	10.1 ± 0.1	1.9 ± 0.1	3.0 ± 0.3	6.0 ± 0.2	9.6 ± 0.2	10.0 ± 0.3

Estimated by: ^a^ ICP-OES; ^b^ Elemental analysis; ^c^ TGA.
